# Protein expression profiles in Meishan and Duroc sows during mid-gestation reveal differences affecting uterine capacity, endometrial receptivity, and the maternal–fetal Interface

**DOI:** 10.1186/s12864-019-6353-2

**Published:** 2019-12-17

**Authors:** Kejun Wang, Kaijie Yang, Qiao Xu, Yufang Liu, Wenting Li, Ying Bai, Jve Wang, Cui Ding, Ximing Liu, Qiguo Tang, Yabiao Luo, Jie Zheng, Keliang Wu, Meiying Fang

**Affiliations:** 1 Department of Animal Genetics and Breeding, National Engineering Laboratory for Animal Breeding, MOA Laboratory of Animal Genetics and Breeding, Beijing key Laboratory for Animal Genetic Improvement, College of Animal Science and Technology, China Agricultural University, Beijing, 100193 People’s Republic of China; 2 College of Animal Science and Veterinary Medicine, Henan Agricultural University, Zhengzhou, 450002 People’s Republic of China; 3 College of Agriculture, Hebei University of Engineering, Handan, 056021 People’s Republic of China

**Keywords:** Protein expression, iTRAQ, Endometrium, Meishan and Duroc pigs

## Abstract

**Background:**

Embryonic mortality is a major concern in the commercial swine industry and primarily occurs early in gestation, but also during mid-gestation (~ days 50–70). Previous reports demonstrated that the embryonic loss rate was significant lower in Meishan than in commercial breeds (including Duroc). Most studies have focused on embryonic mortality in early gestation, but little is known about embryonic loss during mid-gestation.

**Results:**

In this study, protein expression patterns in endometrial tissue from Meishan and Duroc sows were examined during mid-gestation. A total of 2170 proteins were identified in both breeds. After statistical analysis, 70 and 114 differentially expressed proteins (DEPs) were identified in Meishan and Duroc sows, respectively. Between Meishan and Duroc sows, 114 DEPs were detected at day 49, and 98 DEPs were detected at day 72. Functional enrichment analysis revealed differences in protein expression patterns in the two breeds. Around half of DEPs were more highly expressed in Duroc at day 49 (DUD49), relative to DUD72 and Meishan at day 49 (MSD49). Many DEPs appear to be involved in metabolic process such as arginine metabolism. Our results suggest that the differences in expression affect uterine capacity, endometrial matrix remodeling, and maternal-embryo cross-talk, and may be major factors influencing the differences in embryonic loss between Meishan and Duroc sows during mid-gestation.

**Conclusions:**

Our data showed differential protein expression pattern in endometrium between Meishan and Duroc sows and provides insight into the development process of endometrium. These findings could help us further uncover the molecular mechanism involved in prolificacy.

## Background

Litter size is an important economic trait in swine production. Many studies showed that multiple interactive components affect litter size [1, 2], such as uterine capacity [3], ovulation rate [4, 5], and embryonic viability etc. Embryonic mortality accounts for over 30% of the overall mortality in swine herds and remains a challenge to the commercial swine industry [6]. Previous publications mainly focused on early period of gestation because of high fetal mortality ratio. Early embryonic loss before 18 days of gestation primarily due to a failure of one of three critical steps: the switch from maternal to embryonic transcript usage at the four to eight cell stage [7]; blastocyst elongation [8]; or the attachment of conceptuses to the endometrium [9]. Superovulation had been used to increase conceptus number, but was quickly abandoned due to heavy embryonic losses at 30 days of gestation and after [2]. Wilson et al. performed placental efficiency selection in Yorkshire gilts and found that litter size increased and placental weight and piglet weight decreased [3]. Vonnahme et al. reported that there was no association between uterine horn length and conceptus number during early gestation, but found a high positive correlation during middle gestation, and a high association between viable conceptuses and placental weight between day 25 and day 44 of gestation [2, 10]. However, evidence from Lambersons et al. showed selected for placental efficiency did not increase litter size [11]. Thus selections for improving placental efficiency could increase the litter size remain controversial [2, 3, 11, 12]. For further exploring the related factors of litter size, the molecular data is necessary to uncover the genetic mechanism behind.

Chinese Meishan pig is a highly prolific breed, farrowing 3–5 more live piglets per litter than European pig breeds, including the Duroc pig [13], despite a similar ovulation rate [14]. It had been also demonstrated that Chinese Meishan pigs had a 20–34% greater fetal survival than the European pig breeds [15]. Comparisons between the Meishan and other pig breeds indicate that litter size is determined mainly by the recipient females [16, 17]. The larger litter size of Meishan pigs partly results from the changes in the uterine milieu as well as a higher uterine capacity [15]. Evidence from these studies urged us to study the molecular basis of fetal loss during mid-gestation through comparing Meishan and European sows.

Several studies reported that high-throughput transcriptome data were used for identifying the expression differences between sows groups during the early stage of pregnancy [15, 18, 19], which found that there are great change of many genes during the process. However, embryonic loss during mid-gestation (around days 50 to 70 of gestation) were also reported for accounting for 10–15% [6, 15, 20–22] of the total, but till now very few molecular genetic data were collected on sows at this stage of pregnancy for investigating embryonic mortality.

Here, in an effort to identify the molecular mechanisms involved in fetal loss during mid-gestation, we used iTRAQ (isobaric tags for relative and absolute quantification) to globally characterize differentially expressed proteins from endometrial tissues of Meishan and Duroc sows.

## Materials and methods

### Animals and sample collection

All animal procedures used in this study strictly followed protocols approved by Animal Welfare Committee in the State Key Laboratory for Agro-biotechnology at China Agricultural University (Approval number XK257). Six healthy Meishan sows and six healthy Duroc sows were obtained from Shanghai Zhu Zhuang Yuan Company (Shanghai, China) and had been raised in identical conditions. They were randomly selected but were unrelated. All had previously delivered three litters. During the fourth pregnancy, on days 49 and 72 of gestation, three Meishan and three Duroc sows were rendered unconscious by electrical stunning and then immediately bled by cutting the throat. Uteri were picked out and the endometrium around the implantation zones were selected. After removing the obvious blood vessel, around 3 mg tissue was collected for each individual. Fresh tissue was transferred to liquid nitrogen and stored at − 80 °C until use.

### Protein extraction and trypsin digestion

Sample was sonicated three times on ice using a high intensity ultrasonic processor (Scientz) in lysis buffer (8 M urea, 2 mM EDTA, 10 mM DTT and 1% Protease Inhibitor Cocktail). The remaining debris was removed by centrifugation with 20,000 g at 4 °C for 10 mins. Subsequently, the protein was precipitated with 15% cold TCA for 2 h at − 20 °C. After centrifugation at 4 °C for 10 min, the supernatant was discarded. The precipitate was washed twice with cold acetone. Then the protein was redissolved in buffer (8 M urea, 100 mM TEAB, pH 8.0) and the protein concentration was determined with 2-D Quant kit according to the manufacturer’s instructions (GE Healthcare, USA). 100 μg of protein from each sample was digested overnight with trypsin (Promega, USA) using a mass ratio 1:50 (trypsin: protein), followed by second digestion for 4 h (mass ratio 1:100).

### Protein identification and quantitation

Tissues from two animals were used for each breed/pregnancy stage combination, yielding eight independent protein samples. The samples from Meishan pigs on days 72 and 49 were designated MSD72 and MSD49, and samples from Duroc pigs on days 72 and 49 were designated DUD72 and DUD49. iTRAQ labeling was performed using a 6-plex TMT kit (Thermo Scientific, USA) according to the manufacturer’s instructions. iTRAQ labels 127 to 130 were used to tag samples as follows: MSD72:127, MSD49:128, DUD49:129, and DUD72:130. Identical labels were used for the two samples obtained from the same breed and pregnancy stages. Labeled samples were then combined to generate two pools, each pool containing one each of MSD72, MSD49, DUD72, and DUD49.

The pools were then fractionated using high pH reverse-phase HPLC with an Agilent 300Extend C18 column (5 μm particles, 4.6 mm I.D., 250 mm length). A reverse-phase analytical column (Acclaim PepMap RSLC, Thermo Scientific, USA) was used for peptide separation. Peptides were analyzed in a continuous solvent B (0.1% formic acid in 98% acetonitrile) gradient that increased from 7 to 20% over 24 min, 20 to 35% over 8 min, 35 to 80% over 5 min, then held at 80% for 3 min. A constant flow rate of 300 nl/min was maintained on an EASY-nLC 1000 UPLC system. The peptides were analyzed using a Q ExactiveTM hybrid quadrupole-Orbitrap mass spectrometer (Thermo Fisher Scientific, USA). Peptides were subjected to an NSI source, followed by tandem mass spectrometry (MS/MS) in the Q ExactiveTM instrument (coupled online to the UPLC). The Orbitrap was used to detect the intact peptides at a resolution of 70,000. The analysis (one MS scan followed by 20 MS/MS scans) was applied to the top 20 precursor ions above a threshold ion count of 1E4 in the MS survey scan with 30.0 s dynamic exclusion. To prevent overfilling the ion trap, automatic gain control (AGC) was applied. Protein quantitation was calculated as the median ratio of corresponding unique peptides for a given protein. For one replicate, fold change was calculated as the ratio of protein quantity value (computed from unique peptides) of case group to control group. Differentially expressed proteins (DEPs) were identified based on the geometrical mean of the fold change values (calculated from each replicate respectively) for each protein, and two-tail *t-test* was used to compute the *p*-value of significance between groups.

### Bioinformatics analysis

MS/MS data were processed using the Mascot search engine (v.2.3.0) and tandem mass spectra were compared to entries in the Uniprot *Sus scrofa* database (21,047 sequences). Trypsin/P was specified as the cleavage enzyme, allowing up to 2 missing cleavages. Mass error was set to 10 ppm for precursor ions and 0.02 Da for fragment ions. FDR was adjusted to < 1% and the peptide ion score was set > 20. The IDs of identified proteins were converted to UniProt IDs and then GO analysis was performed. Gene Ontology (GO) annotation of the proteome was implemented using the UniProt-GOA database (http://www.ebi.ac.uk/GOA/). InterProScan (http://www.ebi.ac.uk/interpro/) was used to annotate proteins that were absent from the UniProt-GOA database, and proteins were classified using the Gene Ontology annotation tools (http://geneontology.org/). The Kyoto Encyclopedia of Genes and Genomes (KEGG) database was used to annotate protein pathways. A two-tailed Fisher’s exact test was employed to test for enrichment of the differentially expressed proteins relative to all identified proteins.

### Western blotting

Proteins isolated from pig endometrium tissue (extraction steps described above) were used to validate the iTRAQ results. 30 μg of protein was separated by SDS-PAGE and then electro-transferred onto PVDF membrane (Millipore). Membranes were blocked overnight with blocking reagent at 4 °C and then incubated with one of five primary antibodies; CTSB, GLA, CRYAB, DPP4, or ASAH1 (13,000, Abcam) for 2 h at room temperature. Membranes were rinsed six times in TBST (20 mM Tris–Cl, 140 mM NaCl, pH 7.5, 0.05% Tween-20) for 30 min, and then incubated with a secondary antibody (goat-anti rabbit IgG HRP-conjugate, 1:8000, Abmart) for 2 h at room temperature. Membranes were washed again with TBST for 30 min. The membranes of Western blot were incubated with ECL chemiluminescent substrate (ThermoFisher, USA) for 5 min at darkroom. The light output of ECL can be captured using film (Koda, China). Films were imaged with scanner and Image J software (https://imagej.nih.gov/ij/) was used to compare the density of bands. Results are presented as means ±SEM. Differences were tested for statistical significance using ANOVA. *p* < 0.05 was considered the threshold for statistical significance (*, *P* < 0.05; **, *P* < 0.01).

## Results and discussion

The Chinese Meishan pig farrows more live piglets per litter than European pig breeds [13]. Fetal loss appears to be responsible for the difference. The embryonic loss rate is significantly lower in Meishan (~ 14%) than in commercial breeds, including the Duroc (19%~ 39%) [6, 20].

According to our record (three individuals in one group), there is a ~ 13% fetus loss from MSD49 (16.3 ± 0.47) to MSD72 (14.3 ± 0.47), whereas ~ 21% loss from DUD49 (11 ± 0.82) to DUD72 (8.67 ± 0.47) (Additional file 6: Figure S1). Although embryonic loss during mid-gestation (days 50 to 70 of gestation) accounts for 10–15% [6, 20] of the total, genomic studies in sows at this stage of pregnancy have not been done. Comparisons between the Meishan and other breeds indicate that litter size is determined mainly by the recipient females rather than the sire or embryos [18, 19]. We therefore used iTRAQ to compare protein expression profiles in endometrial tissue from Meishan and Duroc sows on days 49 and 72 of pregnancy to identify proteins that are potentially involved in prolificacy differences.

### Classification of proteins identified in endometrial tissue

**Fig. 1:**
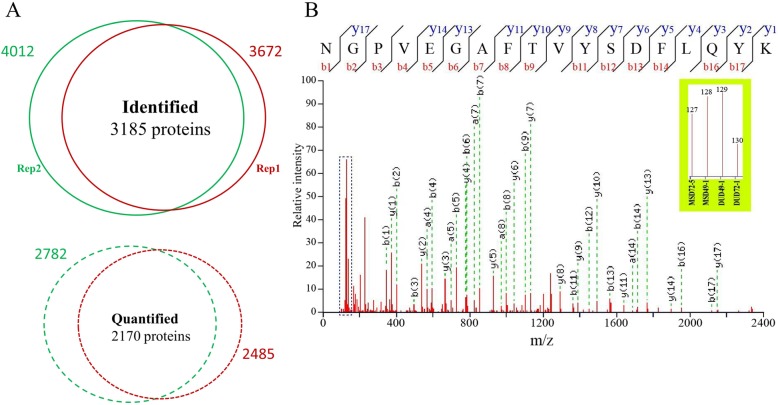
Representative MS/MS spectra and reporter ions for a peptide. Descriptive statistics for proteins identified and quantified in two separate analyses (**a**). The MS/MS spectrum used to identify and quantitate CTSB (**b**). The sequence NGPVEGAFTVYSDFLQYK allows CTSB to be uniquely identified, while the released iTRAQ reporter ions provide the data required for relative quantitation between groups

Proteins from eight animals (two from each of the breed-pregnancy stage groups DUD49, DUD72, MSD49, and MSD72) were labeled, and then analyzed in two independent LC-MS/MS runs. A total of 14,629 and 16,565 unique peptides were identified in the two replicas with a minimum confidence level of 99%, representing 3672 and 4012 proteins, respectively. A substantial number of proteins (3185) were found in both runs (Fig. 1a). In total, 2485 and 2741 proteins were quantified in two independent runs (replicates), of which 2170 proteins were in common and used to compare the relative abundance between groups (Fig. 1a). The common proteins were subjected to GO enrichment analysis. The top ten enriched GO terms are shown in Additional file 7: Figure S2, grouped according to the major GO categories biological process, molecular function, and cellular component. In the biological process category, most proteins are involved in cellular process (GO:0009987), single organism process (GO:0044699), metabolic process (GO: 0008152), and single organism cellular process (GO: 0044763). Within the molecular function category, most proteins participated in binding (GO:0005488), catalytic activity (GO: 0003824), organic cyclic compound binding (GO:0097159), and heterocyclic compound binding (GO:1901363). Finally, for the cellular component category, most proteins were found in cell (GO:0005623), cell part (GO:0044464), intracellular (GO:0005622), and intracellular part (GO:0044424).

### Identification and validation of DEPs

Fold change was calculated by comparing the median ratio of corresponding peptides of a given protein for each replicate. Representative MS/MS spectra and reporter ions derived from the differentially expressed protein CTSB are shown in Fig. 1b. Differentially expressed proteins (DEPs) were identified based on the geometrical mean of the fold change value calculated for each protein in the two replicates. Using 1.3/0.70 (*p*-value< 0.05) as mean value thresholds to classify proteins as increased or decreased, we identified DEPs between DUD72 vs. DUD49, MSD72 vs. MSD49, MSD49 vs. DUD49, and MSD72 vs. DUD72 (Table 1). Replicate samples yielded results that were highly similar (Additional file 8: Figure S3).

**Table 1:** Descriptive statistics for differentially expressed proteins

Group	Increased	Decreased	Total
DUD72 vs. DUD49	35	79	114
MSD72 vs. MSD49	43	27	70
MSD49 vs. DUD49	45	69	114
MSD72 vs. DUD72	56	42	98

**Fig. 2:**
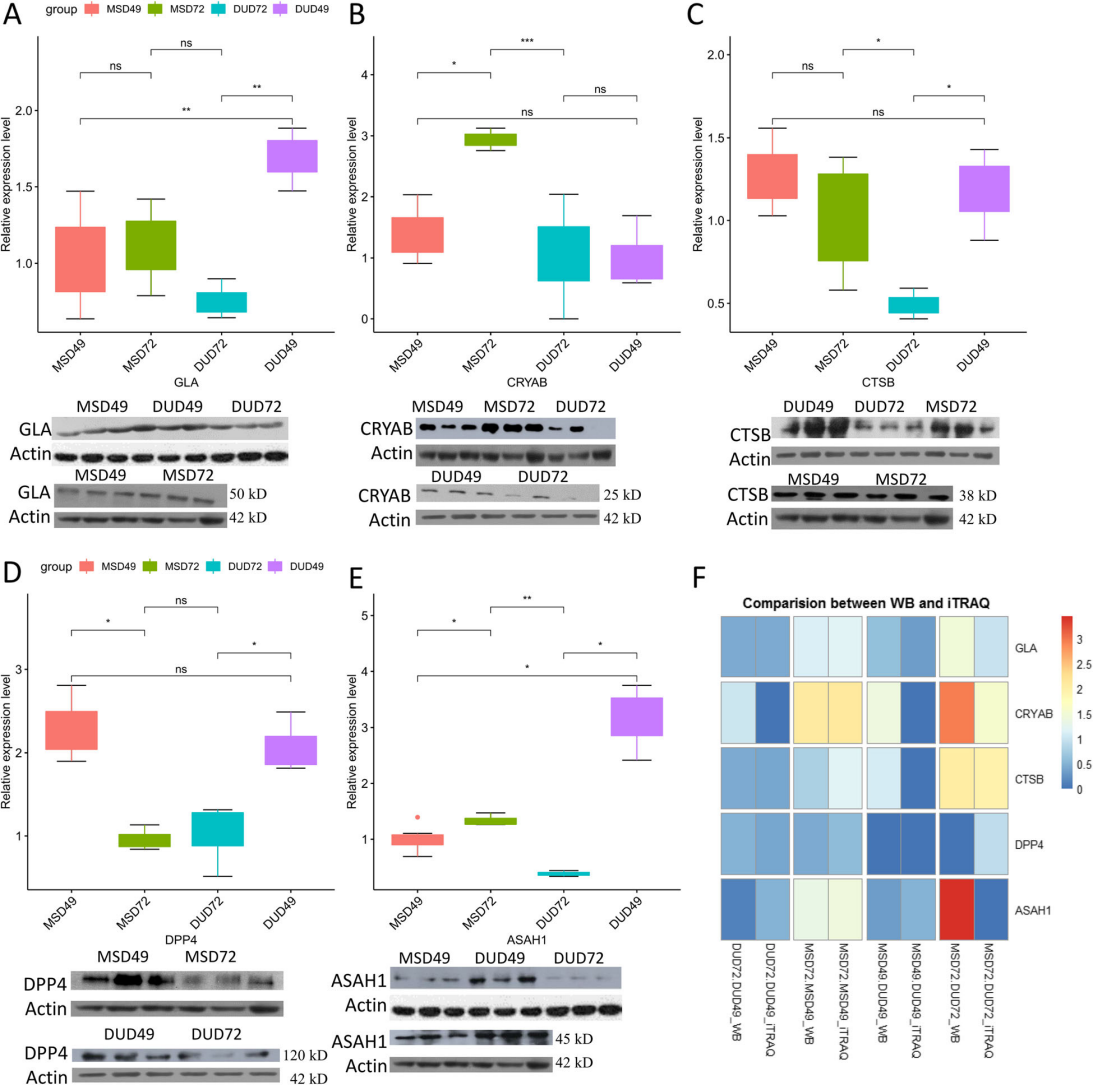
Western blot validation for five DEPs. Based on band intensity, the relative expression of five proteins was adjusted by housekeeping Actin protein and then normalized to compare. GLA (**a**), CRYAB (**b**), CTSB (**c**), DPP4 (**d**), and ASAH1 (**e**). *, *P* < 0.05; **, *P* < 0.01. Three lanes represent the three biological repeats in one group. Heatmap comparing average fold change in expression of the five genes as measured by western blot and iTRAQ (**f**). Missing values were set to zero

**Fig. 3:**
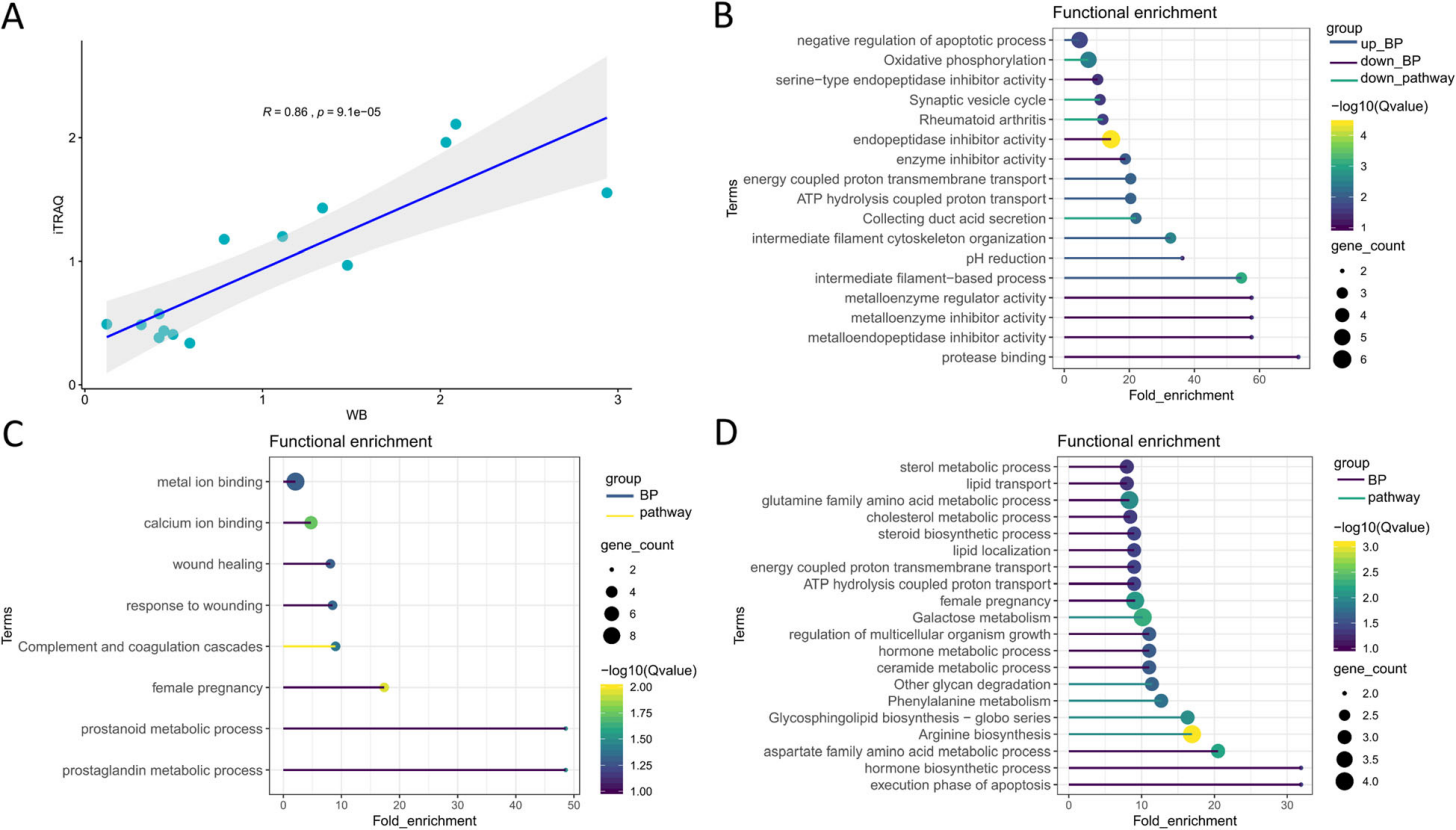
Correlation and functional enrichment analysis. Correlation analysis showing that the changes in expression for five DEPs are consistent between WB and iTRAQ (**a**). The top biological processes and pathways enriched by DEPs from MSD72 vs. MSD49 (**b**). Top biological process and pathways enriched by increased (**c**) and decreased (**d**) DEPs from DUD72 vs. DUD49

Five differentially expressed proteins (GLA, CRYAB, CTSB, ASAH1, and DPP4) were randomly selected and quantitated by western blot to test the reliability of the iTRAQ analysis (Fig. 2a-e). The western blot results for all five proteins were consistent with the iTRAQ analysis. The changes in expression levels, as measured by the two methods, are compared in Fig. 2f. The correlation between the fold change values is 0.86 (*p* = 9.1e-05) (Fig. 3a), supporting the conclusion that the iTRAQ analysis reliably identifies DEPs.

### Differential protein expression during pregnancy in Meishan and Duroc pigs

To further characterize protein expression during the two points of mid-late stage pregnancy, DEPs were identified by comparing expression on days 49 and 72 within each breed. The DEPs were then subjected to functional enrichment analysis. For Meishan pigs (MSD72 vs. MSD49), we found 43 increased and 27 decreased proteins (Table 1). The DEPs and corresponding functional enrichment analyses are shown in Additional file 1: Table S1. Terms associated with GO biological processes (six for increased and seven for decreased proteins) and KEGG pathways are presented in Fig. 3b. Several GO terms associated with increased DEPs were of potential interest, such as intermediate filament cytoskeleton and intermediate filament-based process. Four KEGG pathways were also associated with the increased DEPs but were not as informative. GO terms associated with the decreased DEPs included endopeptidase inhibitor activity, serine-type endopeptidase inhibitor activity, metalloendopeptidase inhibitor activity, and extracellular vesicle. In contrast, the decreased DEPs were not significantly enriched in any KEGG pathway.

The comparison in Duroc pigs (DUD72 vs. DUD49) identified 35 increased and 79 decreased DEPs (Additional file 2: Table S2). Functional enrichment analysis results are summarized for each DEP in Additional file 2: Table S2. Of potential interest are the biological process terms female pregnancy and prostanoid metabolic process. Only one significant pathway, complement and coagulation cascades, was enriched by the increased DEPs (Fig. 3c). The top fifteen biological processes and five pathways enriched by the decreased DEPs are shown in Fig. 3d. Of potential interest are terms describing several metabolic processes (such as sterol, lipid, cholesterol, galactose, glutamine, fatty acid), female pregnancy, arginine biosynthesis, and arginine and proline metabolism.

DEPs were also identified by comparison between breeds. Only 7 DEPs were in common with those found by the within-breed comparisons described above. Two proteins, CNN1 and TRIM29, were identified in the increased DEPs from MSD72 vs MSD49 and DUD72 vs DUD49. DPP4 and ANXA10 were identified in the decreased DEPs from MSD72 vs MSD49 and DUD72 vs DUD49. Three proteins, PODN, ASAH1 and CPS1, exhibited differential reverse expression patterns between Meishan and Duroc pigs during mid-pregnancy.

### Functional clustering of DEPs at days 49 and days 72

**Fig. 4:**
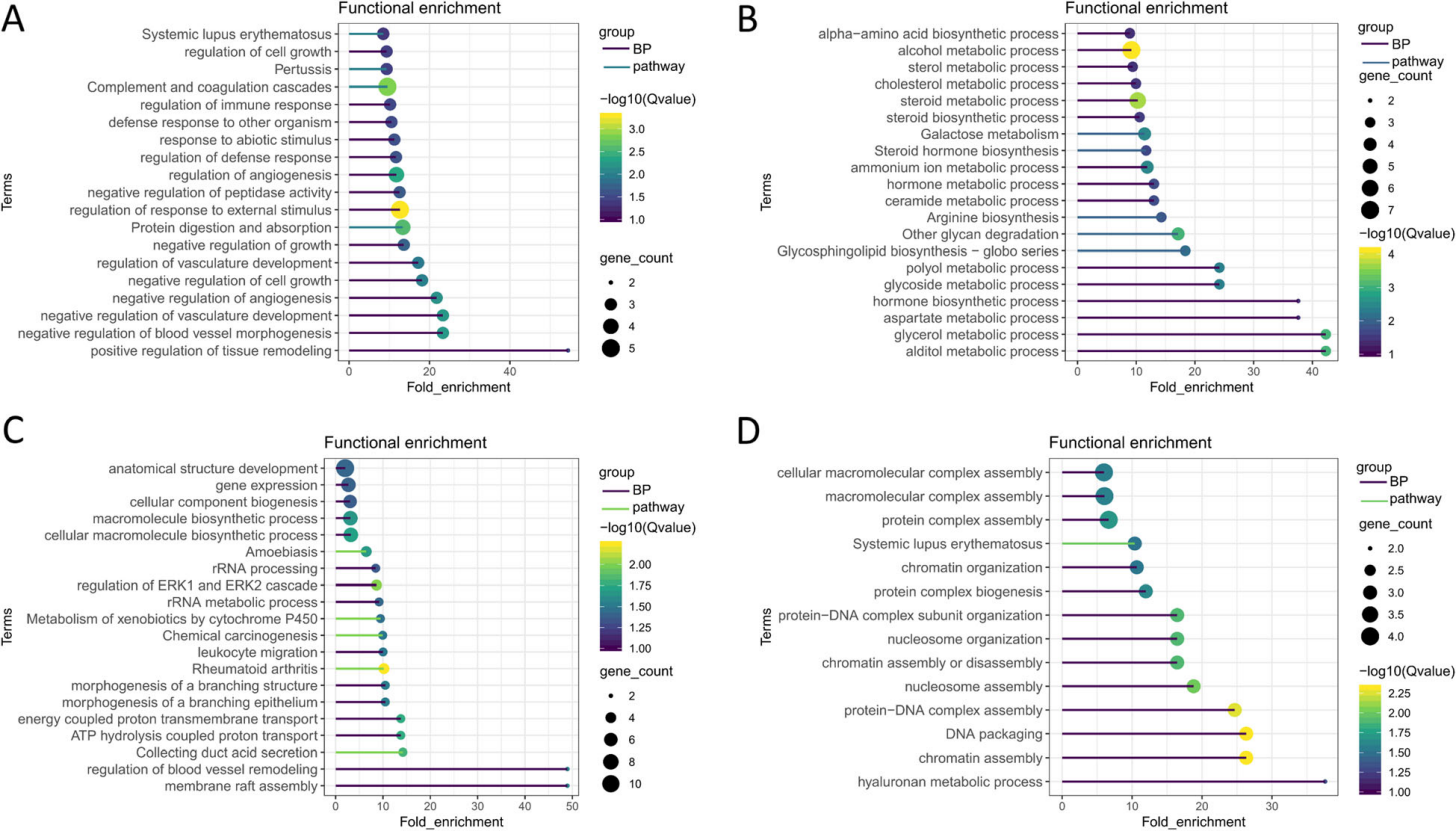
Functional enrichment analysis for DEPs between Meishan and Duroc sows at days 49 and days 72. The top fifteen biological processes and five pathways enriched by increased (**a**) and decreased (**b**) DEPs from MSD49 vs. DUD49. Top fifteen biological processes and five pathways enriched by increased (**c**) and decreased (**d**) DEPs from MSD72 vs. DUD72

To characterize the differences in endometrium protein profiles between Meishan and Duroc pigs, proteins from each developmental stage were compared, and then the DEPs were subjected to functional enrichment analysis. The top fifteen biological process and five pathway terms are presented in Fig. 4.

At day 49, we identified 114 DEPs (MSD49 vs. DUD49), consisting of 45 increased and 69 decreased DEPs (Additional file 3: Table S3). The DEPs are associated with several potentially interesting GO biological process terms, such as regulation of immune response, angiogenesis, and tissue remodeling (Fig. 4a). The pathway analysis suggests that the DEPs may be involved in immune-related disease processes. Most of decreased DEPs were associated with metabolic and biosynthetic terms, including sterol metabolism, glycoside metabolism, cholesterol metabolism, and steroid biosynthetic process (Fig. 4b). Enriched pathways included galactose metabolism, steroid hormone biosynthesis, and arginine biosynthesis.

At day 72, 98 DEPs (56 increased and 42 decreased) were identified between the two breeds (Additional file 4: Table S4). Figure 4c and d show the results of the functional enrichment analyses for increased and decreased DEPs. Increased proteins were associated with GO terms such as extracellular matrix component, regulation of ERK1 and ERK2 cascade, and hydrogen ion transmembrane transporter activity, and were associated with pathways involved in oxidative phosphorylation, metabolism of xenobiotics by cytochrome P450, and rheumatoid arthritis (Fig. 4c). Decreased proteins were associated with the GO terms serine-type endopeptidase inhibitor activity, DNA packaging complex, mucleosome organization, and hyaluronan metabolic process (Fig. 4d).

### Differential expression proteins are related to uterine capacity

**Fig. 5:**
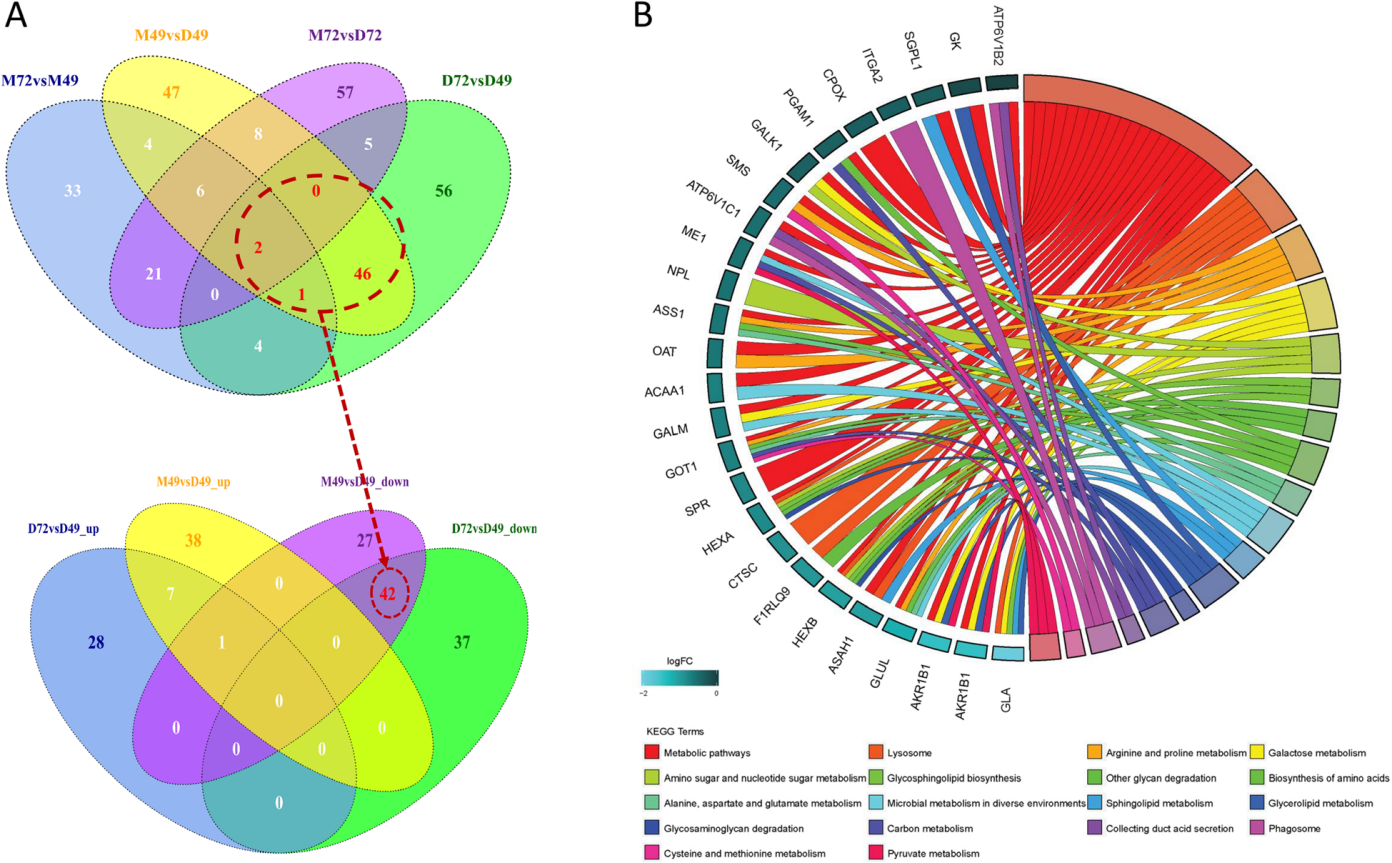
Identification and functional enrichment analysis of shared DEPs from comparisons of MSD72 vs. MSD49, MSD49 vs. DUD49, MSD72 vs. DUD72, and DUD72 vs. DUD49. The Venn diagram shows unique and shared DEPs associated with the four comparisons (**a**). Increased or decreased DEPs from MSD49 vs. DUD49 and DUD72 vs. DUD49 are compared in the bottom panel. The red dotted line connects the 42 decreased DEPs that are shared between the two groups. Results of functional enrichment analysis for the 42 shared DEPs (**b**). The curved lines connect GO categories with their associated DEPs. The color shading in the DEP boxes is proportional to the DEP fold change values, which were obtained from MSD49 vs. DUD49 and then transformed into logFC values (log2 fold change)

To analyze the expression patterns of the two breeds in more detail, the DEPs obtained from analyses of MSD72 vs. MSD49, MSD49 vs. DUD49, MSD72 vs. DUD72, and DUD72 vs. DUD49 were compared to identify commonalities and differences. The comparison between MSD49 vs. DUD49 and DUD72 vs. DUD49 revealed 49 proteins in common, of which 42 DEPs were classified as decreased (Fig. 5a). The common proteins were then subjected to functional enrichment analysis. A total of eighteen KEGG pathways were enriched, most of which were metabolic pathways (Fig. 5b), including pathways for arginine and proline, galactose, glycerolipids, cysteine and methionine, and amino sugars and nucleotide sugars. The analysis shows that many proteins involved in metabolic pathway were highly expressed in DUD49 relative to both MSD49 and DUD72. The result suggests that higher energy absorption and utilization occur in DUD49, potentially associated with higher fetal growth. Meishan conceptuses are significantly smaller than other commercial breeds (including Duroc) from Europe [23, 24] and Americas [25]. One possible interpretation is that excessive fetal growth leads to an overcrowded uterine environment, which reduces uterine capacity and increases fetal loss [26, 27].

The arginine metabolism pathway was enriched by the 42 overlapping DEPs (Fig. 5a-b). Arginine is an important amino acid involved in pig reproductive functions [28]. Arginine participates in muscle tissue protein synthesis, and is a precursor of biologically active molecules promoting embryonic and fetal growth and development [29, 30]. Arginine is an important factor in fetal growth, although reports describing the benefits of arginine supplementation are contradictory [30]. Many studies show that excess arginine supplementation has a negative effect on embryo survival and accelerates fetal growth, which in turn leads to an overcrowded uterine environment. In our study, higher levels of proteins related to arginine metabolism were observed in DUD49 relative to MSD49 (Fig. 5 and Additional file 3: Table S3). The increase may contribute to the generally larger fetal body size of Durocs relative to Meishans [31], consistent with previous reports that Meishan conceptuses are significantly smaller than in other breeds from Europe [23, 24] and Americas [25].

We also identified six increased proteins (KRT18, ACTC1, DES, SYNM, CRYAB, and PRPH) involved in intermediate filament cytoskeleton organization and contractile fiber formation in the MSD72 vs. MSD49 comparison (Additional file 1: Table S1 & Fig. 3b). In humans, the expression of intermediate filaments in endometrial glands changes with the onset of pregnancy and in endometriosis [32]. In the pig endometrium, intermediate filaments are involved in mechanisms responsible for elongation of conceptuses, and for adaptive responses of the uterine wall to the increasing mechanical forces due to fetal growth and accumulation of fetal fluids [33]. KRT18 encodes the type I intermediate filament chain cytokeratin 18 [34] and is expressed in the luminal and glandular epithelium of human and rabbit endometrium during pregnancy [35]. CRYAB is a small heat shock protein that acts as a chaperone that primarily binds misfolded proteins, such as desmin and cytoplasmic actin, and helps to maintain cytoskeletal integrity [36]. To increase uterine capacity, the most obvious strategy is to increase uterine size [31]. Uterine capacity may be increased by selecting for increased uterine length, based on the strong genetic correlation between uterine length and uterine capacity [37]. Additionally, it has been reported that Meishan pigs possess relatively greater uterine capacity than European pigs through retarding fetal growth [31]. Therefore, the proteins that are expressed at different levels in Meishan and Duroc sows might be responsible for enlarging uterine size and retarding fetal growth rate, accounting for the differences in embryonic loss between the two breeds.

### Differentially expressed proteins are involved in endometrial receptivity and maternal-fetal interface process

Extracellular matrix remodeling is necessary for endometrial receptivity and early implantation processes, such as secretory transformation, implantation, and decidualization [38]. Three protease families, the cysteine, serine, and matrix metalloproteinases, are involved in endometrial matrix degradation early in implantation [39]. The difference in endometrial structural modification is the main reason for the functional difference between Meishan and Yorkshire uteri [15, 40, 41]. The process of extracellular matrix remodeling is represented by three protein categories: extracellular matrix component, proteases, and proteases catalyzer. In this study, we identified six increased proteins in DUD72 vs. DUD49 (LAMB2, TGFBI, HAPLN1, COL3A1, CLU and FBN1) that clustered in the extracellular matrix (Additional file 2: Table S2). These proteins are components of the extracellular matrix [42, 43], and are significantly increased in the endometrium at the time of implantation, suggesting a role in blastocyst attachment [44]. Furthermore, some cathepsin proteases (CTSB, CTSC, and CTSZ) were decreased in DUD72 vs. DUD49 (Additional file 2: Table S2). CTSB, CTSC, and CTSZ are involved in the degradation of extracellular matrix proteins including laminin, fibrilin, and collagen [45]. In contrast, no significant differences in the levels of these proteins were observed in MSD72 vs. MSD49. However, three additional extracellular matrix proteins, COL4A2, COL4A1, and LAMB1, were expressed at higher levels in MSD72 vs. DUD72 (Additional file 4: Table S4). CTSB protein was highly expressed in MSD72 relative to DUD72 (Additional file 4: Table S4). CTSB is mainly responsible for degradation of Type IV collagen (including COL4A2 and COL4A1) and laminin (including LAMB1, 45]. Although proteases are responsible for endometrium matrix remodeling, protease activities are affected by secretion of enzyme inhibitors that are required to protect the endometrium from invasion [46]. We found higher levels of CST3, an inhibitor of CTSB activity [47], in MSD72 vs. DUD72, while other protease inhibitors (SLPI, ITIH1, ITIH2, UFAP, UFBP, and SERPING1) were highly expressed in DUD72 vs. MSD72. These proteases inhibitors were all enriched in the GO term serine proteases inhibitor (Additional file 4: Table S4). We also observed that the metalloprotease inhibitors SLPI, SERPING1, FETUB, TIMP, and ITIH1 decreased in MSD72 vs. MSD49 (Additional file 1: Table S1). These data suggest that different proteases and protease inhibitors control the remodeling of the endometrium extracellular matrix during mid-gestation in Meishan and Duroc sows.

Embryonic-maternal cross-talk is an important mechanism for successful pregnancy, conceptus growth, and viability [48, 49]. Maternal-embryo cross-talk is first detected in the implantation window [50] and continues throughout the pregnancy process. Exosomes, which are cell-derived vesicles, function as a communicating language facilitating this process [51, 52]. In this study, enriched GO terms such as extracellular vesicular exosome and membrane-bounded vesicle were associated with many DEPs detected in the proteomic comparison between Meishan and Duroc sows during pregnancy. The comparison between MSD72 and MSD49, 17 increased proteins and 12 decreased proteins were detected to be associated with the extracellular vesicular exosome (Additional file 5: Table S5). This was also the case for 31 decreased proteins detected in DUD72 vs. DUD49 comparison group (Additional file 5: Table S5). It is very interesting that ASAH1 was increased in the former comparison group, but decreased in the latter comparison group (Additional file 1: Table S1 and Fig. 2e). It is already known that ASAH1 plays a crucial role in regulating cell proliferation, migration, and angiogenesis [53]. DPP4 levels decreased at day 72 relative to day 49 in both Meishan and Duroc sows, but the decrease was more pronounced in Duroc sows (Additional file 1: Table S1 and Fig. 2d). DPP4 is a differentiation marker in human endometrial glandular cells [54]. Together, these data suggest that the level of maternal-embryo cross-talk differs between Duroc and Meishan sows in the middle of pregnancy. We hypothesize that this factor might contribute to the differences in prolificacy between Meishan and Duroc sows. This study shows differential protein expression pattern in endometrium between Meishan and Duroc sows during mid-gestation, and might provide evidence for elucidating the differences in embryonic loss between two breeds. However, it cannot be excluded that our results might be bias because of two biological replicates used. Previous publication showed that few biological repeats will affect the call rate of differential proteins with low expression and the true positive rate of results. Significant results in the study may be due to biological variation, the expression patterns are only specific to the investigated individuals and may not a characteristic of the study population. More samples will be needed for further identification of the differential proteins and its function analysis. In addition, the size and weight of the fetuses are also related to embryo loss according to our data and previous publications [24, 25], which should also be included for further understanding of the genetic mechanism of embryo loss.

## Conclusion

In summary, we found differences in the protein expression pattern in endometrium tissue derived from Meishan and Duroc sows during mid-gestation and provided insight into the development process of endometrium. These findings could help us further uncover the molecular mechanism involved in prolificacy.

## Supplementary information

**Additional file 1: Table S1.** List of DEPs and functional enrichment analysis results for DEPs in Meishan sows.

**Additional file 2: Table S2.** List of DEPs and functional enrichment analysis results for DEPs in Duroc sows.

**Additional file 3: Table S3**. List of DEPs and functional enrichment analysis results for DEPs at day 49.

**Additional file 4: Table S4.** List of DEPs and functional enrichment analysis results for DEPs at day 72.

**Additional file 5: Table S5.** List of DEPs associated to extracellular vesicular exosome.

**Additional file 6: Figure S1.** Comparison of fetus number between days 49 and days 72 of Duroc and Meishan sows.

**Additional file 7: Figure S2.** Functional enrichment analyses for identified proteins common to two separate runs. Biological process (A); cellular component (B); molecular function (C).

**Additional file 8: Figure S3.** Correlation of DEPs between two replicates in four groups.

## Data Availability

All relevant information supporting the results of this paper are included within the article and its additional files. Protein sequencing data will be available from the corresponding author upon reasonable request.

## Abbreviations

ACTC1: Cardiac muscle alpha actin 1
ANXA10: Annexin A10
ASAH1: Acid ceramidase
CLU: Clusterin
CNN1: Calponin-1
COL3A1: Collagen alpha-1(III) chain preproprotein
COL4A1: Collagen alpha-1(XIV) chain
COL4A2: Collagen alpha-2(IV) chain
CPS1: Carbamoyl-phosphate synthase
CRYAB: Alpha-crystallin B chain
CST3: Cystatin-C
CTSB: Cathepsin B
CTSC: Pept_C1 domain-containing protein
CTSZ: Cathepsin Z
DEPs: Differentially expressed proteins
DES: Desmin
DTT: DL-Dithiothreitol
EDTA: Ethylenediaminetetraacetic acid
FBN1: Fibrillin-1
FETUB: Fetuin-B
GO: Gene Ontology
HAPLN1: Hyaluronan and proteoglycan link protein 1
ITIH1: Inter-alpha-trypsin inhibitor heavy chain H1
ITIH2: Inter-alpha-trypsin inhibitor heavy chain H2
iTRAQ: Isobaric tags for relative and absolute quantification
KEGG: Kyoto Encyclopedia of Genes and Genomes
KRT18: Keratin, type I cytoskeletal 18
LAMB1: Laminin subunit beta-1
LAMB2: Laminin subunit beta-2
PAGE: Polyacrylamide gel electrophoresis
PODN: Podocan
PRPH: Peripherin-2
PVDF: Poly (vinylidene fluoride)
SERPING1: Plasma protease C1 inhibitor
SLPI: Antileukoproteinase
SYNM: Synemin
TGFBI: Transforming growth factor-beta-induced protein ig-h3
TIMP: Metalloproteinase inhibitor 1
TRIM29: Tripartite motif-containing protein 29
UFAP: Uteroferrin-associated protein
