# Supplementary figures and images for: Protein expression profiles in Meishan and Duroc sows during mid-gestation reveal differences affecting uterine capacity, endometrial receptivity, and the maternal–fetal Interface
Source: BMC Genomics. 2019 Dec 17;20:991. doi: 10.1186/s12864-019-6353-2 (PMC6918595; doi:10.1186/s12864-019-6353-2)

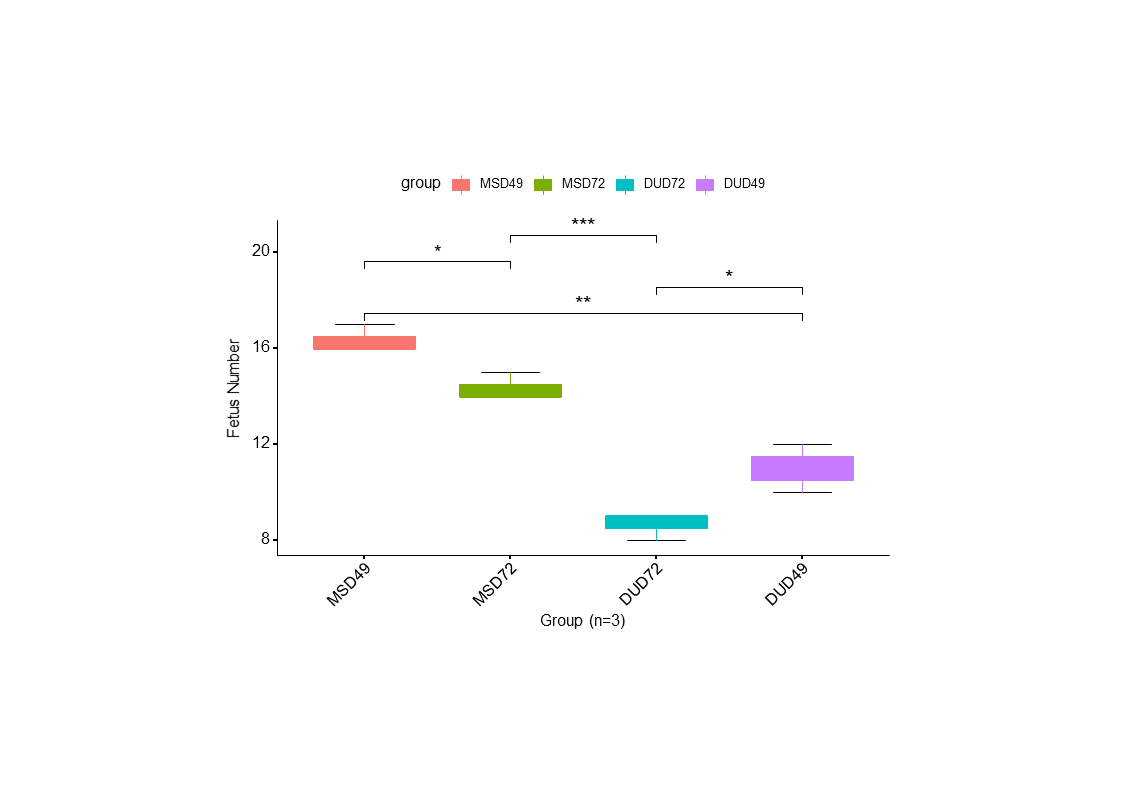

Supplement: Supplementary file 6 — Additional file 6: Figure S1. Comparison of fetus number between days 49 and days 72 of Duroc and Meishan sows. [file 12864_2019_6353_MOESM6_ESM.png]

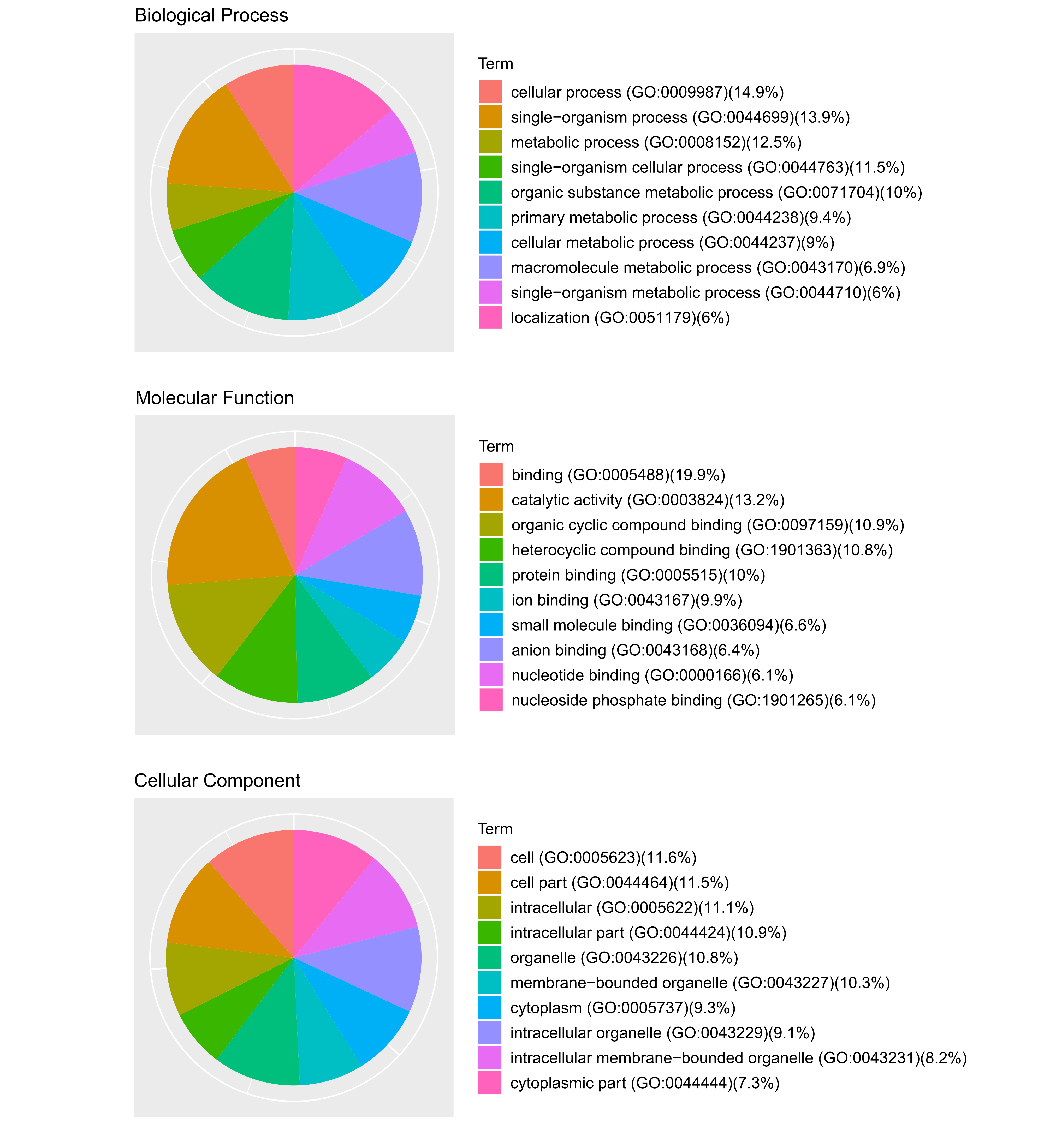

Supplement: Supplementary file 7 — Additional file 7: Figure S2. Functional enrichment analyses for identified proteins common to two separate runs. Biological process (A); cellular component (B); molecular function (C). [file 12864_2019_6353_MOESM7_ESM.png]

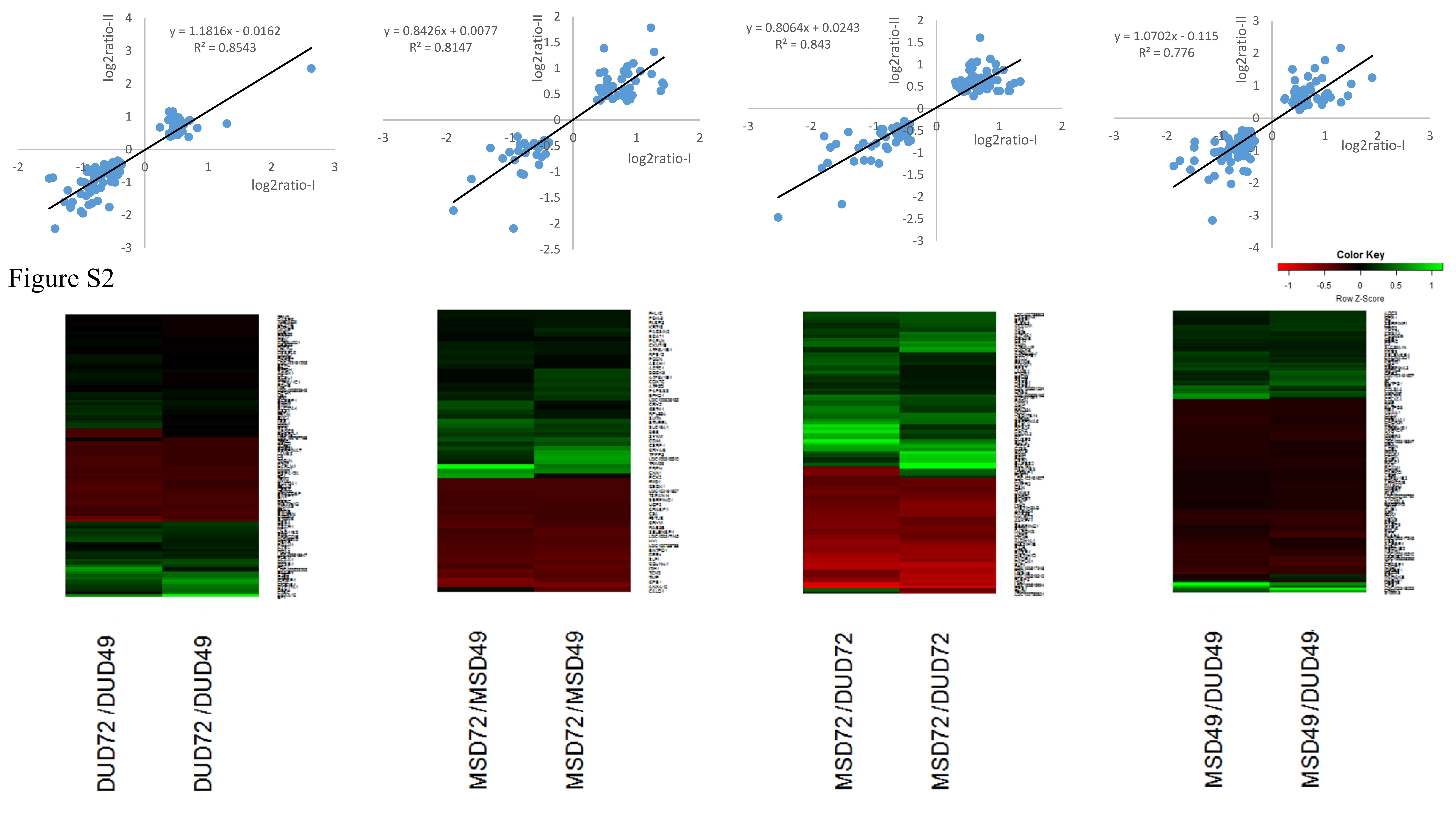

Supplement: Supplementary file 8 — Additional file 8: Figure S3. Correlation of DEPs between two replicates in four groups. [file 12864_2019_6353_MOESM8_ESM.jpg]
